# Supplementary figures and images for: Cytoplasmic dynein-1 cargo diversity is mediated by the combinatorial assembly of FTS–Hook–FHIP complexes
Source: eLife. 2021 Dec 9;10:e74538. doi: 10.7554/eLife.74538 (PMC8730729; doi:10.7554/eLife.74538)

CTRL  
FHIP1A  
FHIP1B  
FHIP2A  
FHIP2B  
Hook2

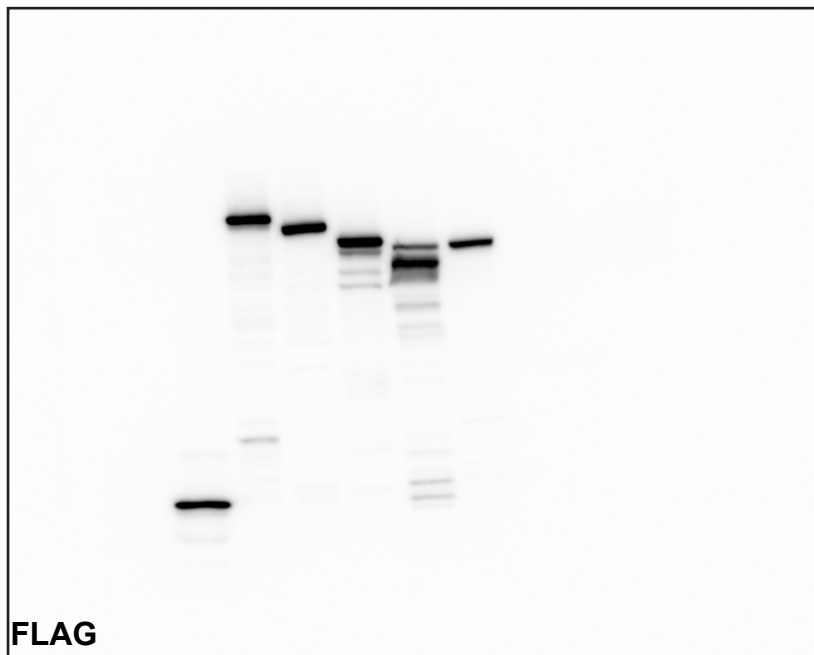

Raw image: Fig1supp1A.scn

Supplement: Figure 1—figure supplement 1—source data 1. [file elife-74538-fig1-figsupp1-data1.pdf]

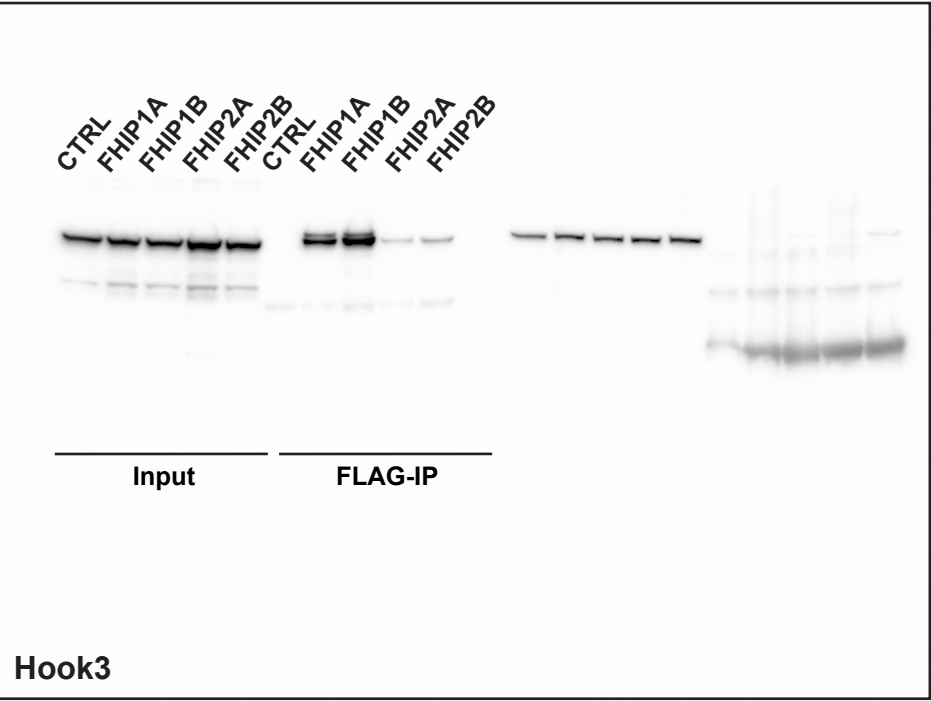

Raw image: Fig2B\_Hook3.scn

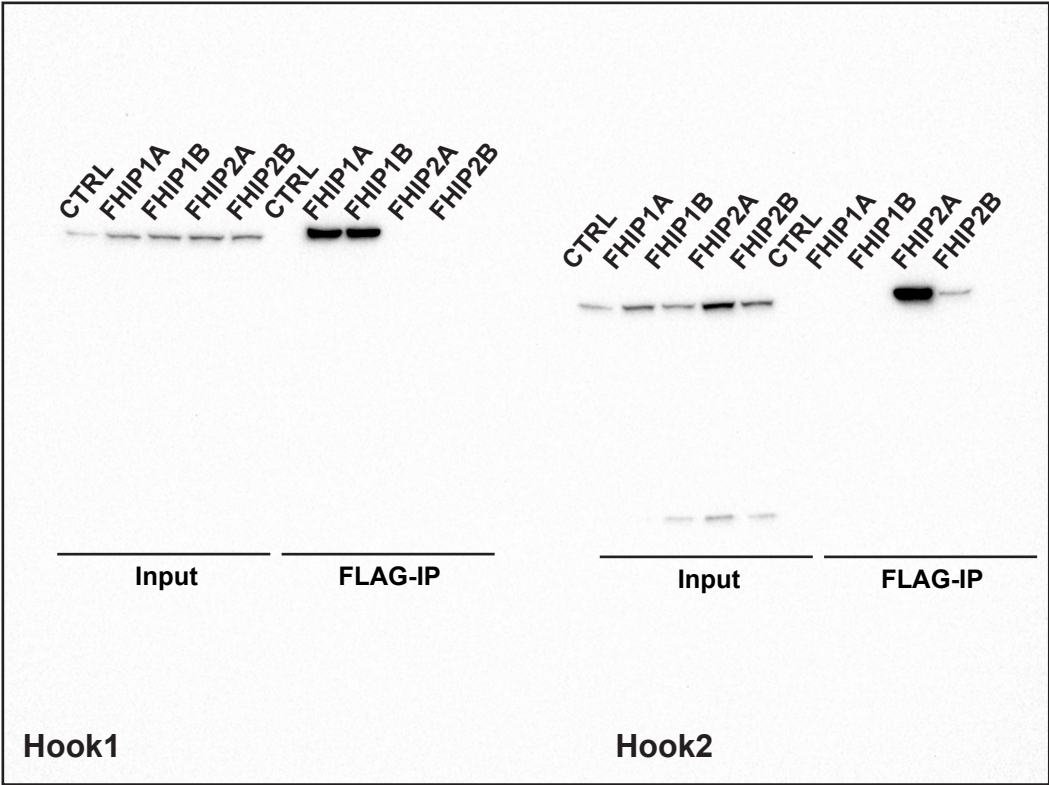

Raw image: Fig2B\_Hook1\_Hook2.scn

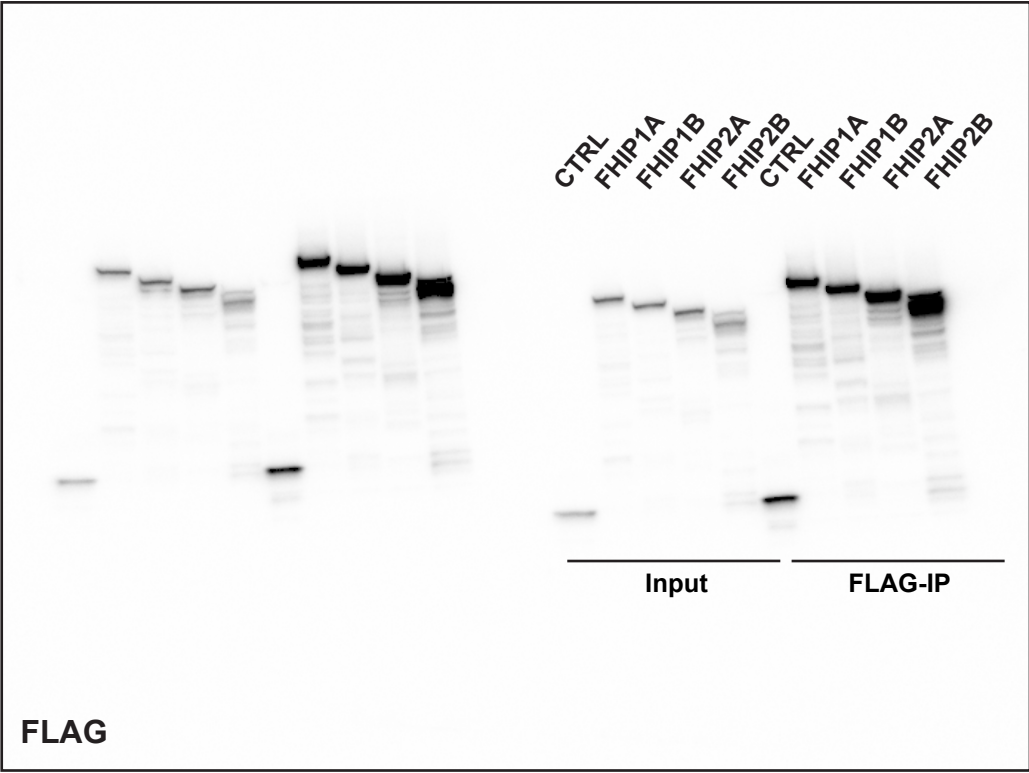

Raw image: Fig2B\_FLAG.scn

Supplement: Figure 2—source data 1. — Relevant lanes are marked on the images. [file elife-74538-fig2-data1.pdf]

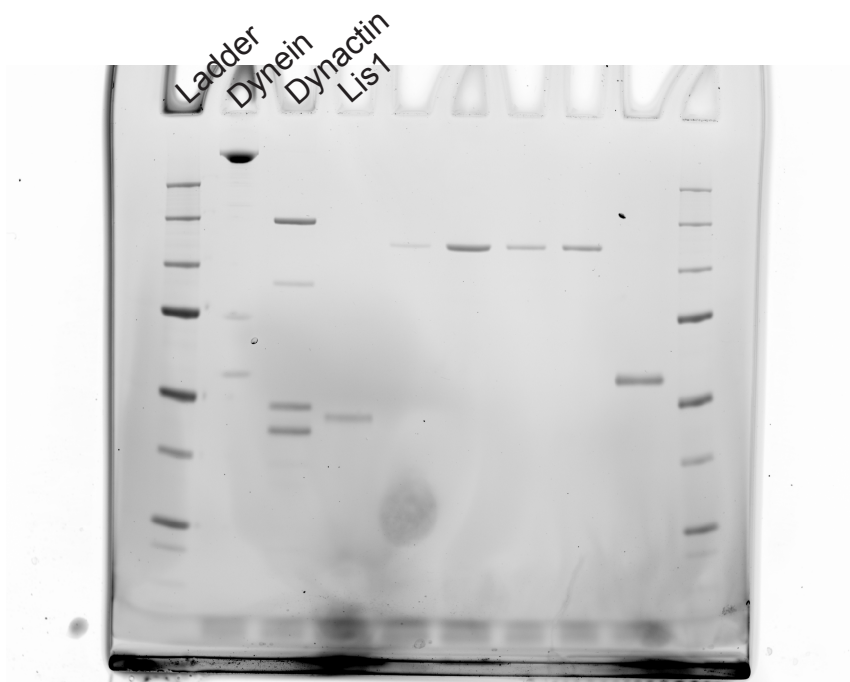

Raw image: Fig3supp1left.scn

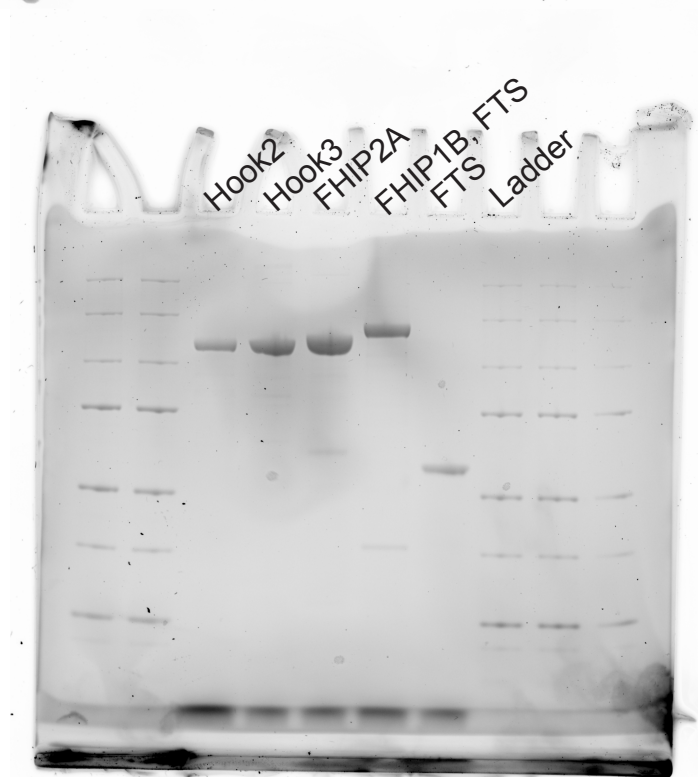

Raw image: Fig3supp1right.scn

Supplement: Figure 3—figure supplement 1—source data 1. — Relevant lanes are marked on the images. [file elife-74538-fig3-figsupp1-data1.pdf]

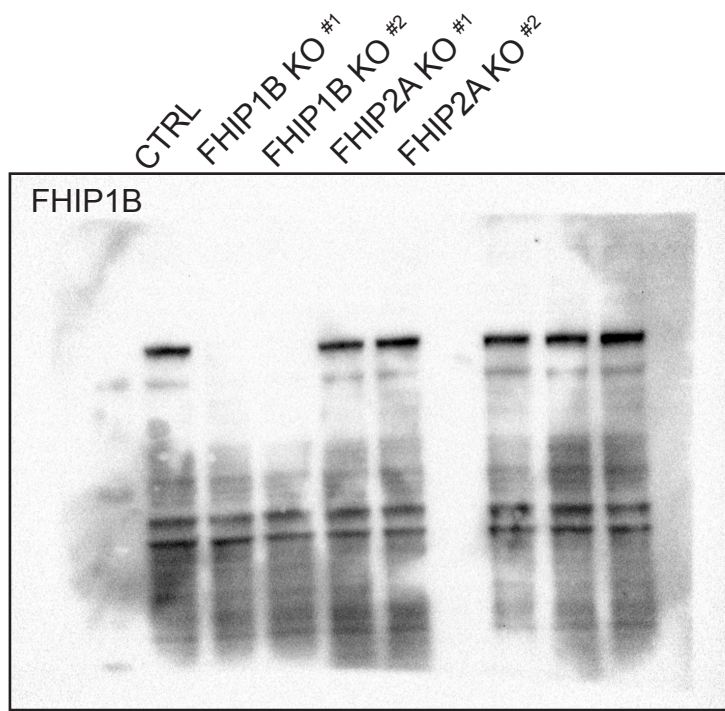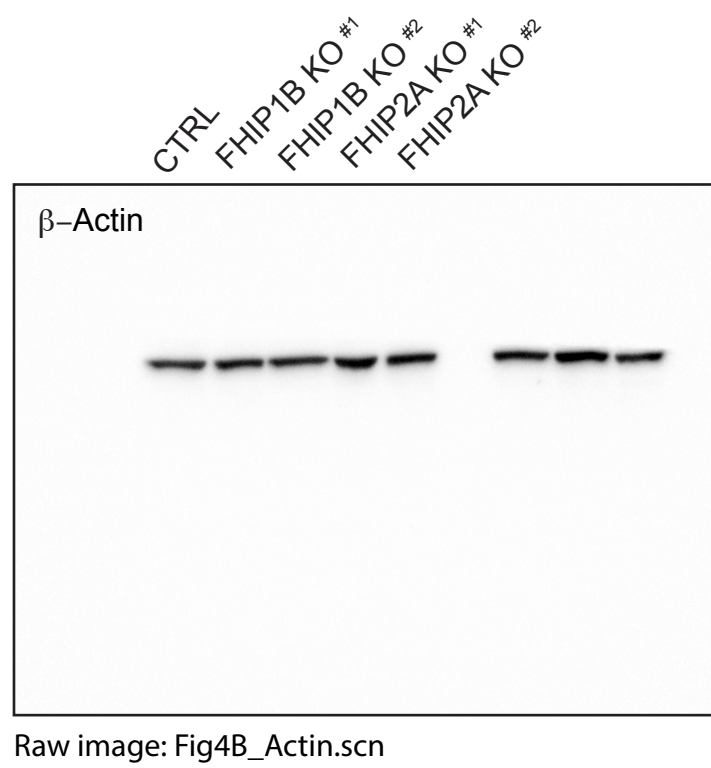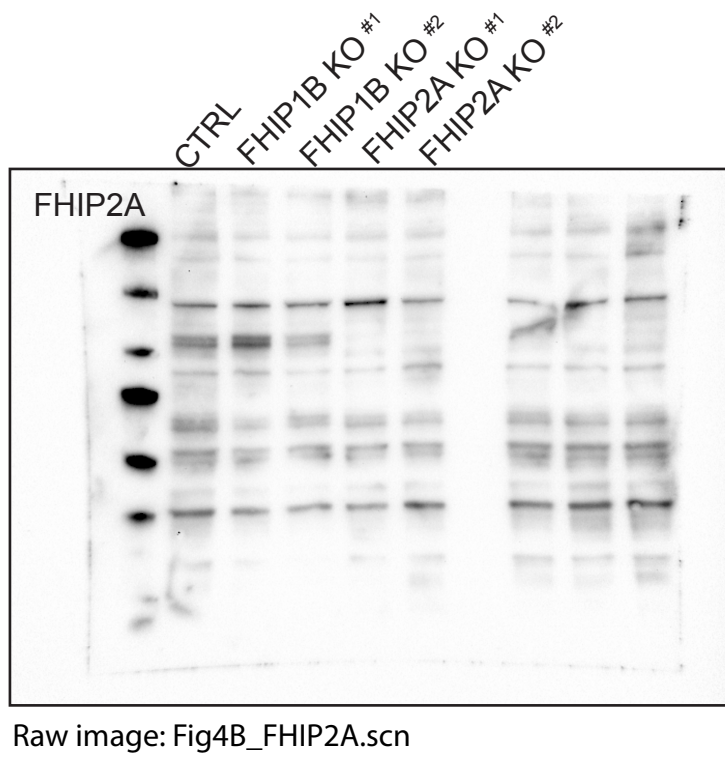

Supplement: Figure 4—source data 1. — Relevant lanes are marked on the images. [file elife-74538-fig4-data1.pdf]
