# Supplementary material for: Cytoplasmic dynein-1 cargo diversity is mediated by the combinatorial assembly of FTS–Hook–FHIP complexes
Source: eLife. 2021 Dec 9;10:e74538. doi: 10.7554/eLife.74538 (PMC8730729; doi:10.7554/eLife.74538)
Supplement: Figure 2—figure supplement 1—source data 1. — Relevant lanes are marked on the images. [file elife-74538-fig2-figsupp1-data1.pdf]

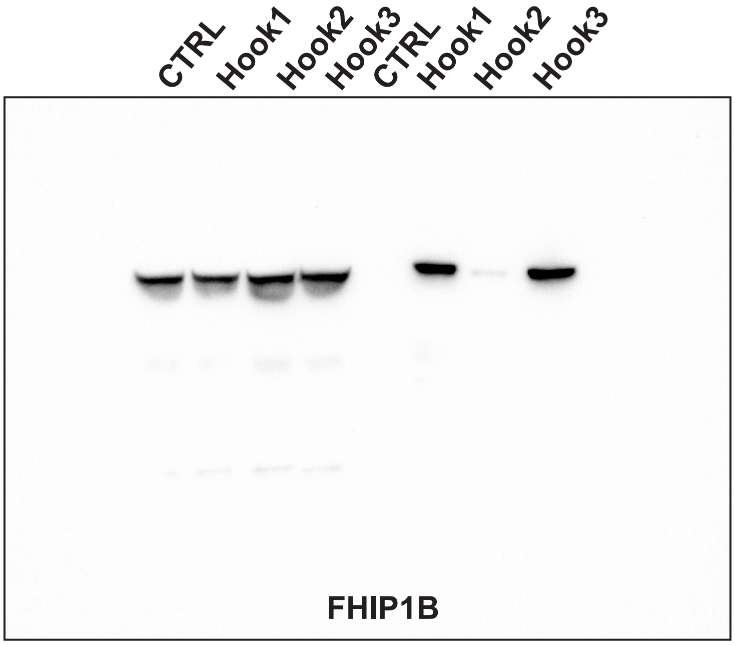

Raw image: Fig2supp1_FHIP1B.scn

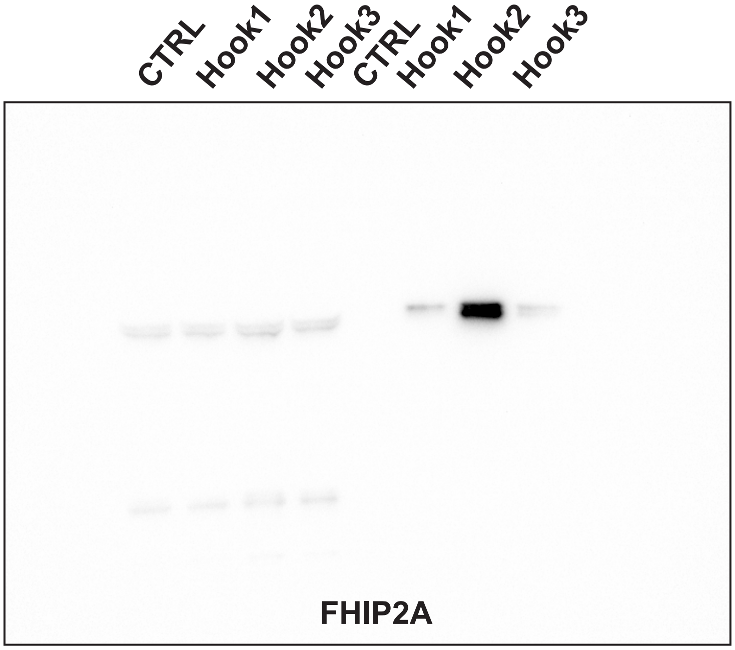

Raw image: Fig2supp1_FHIP2A.scn

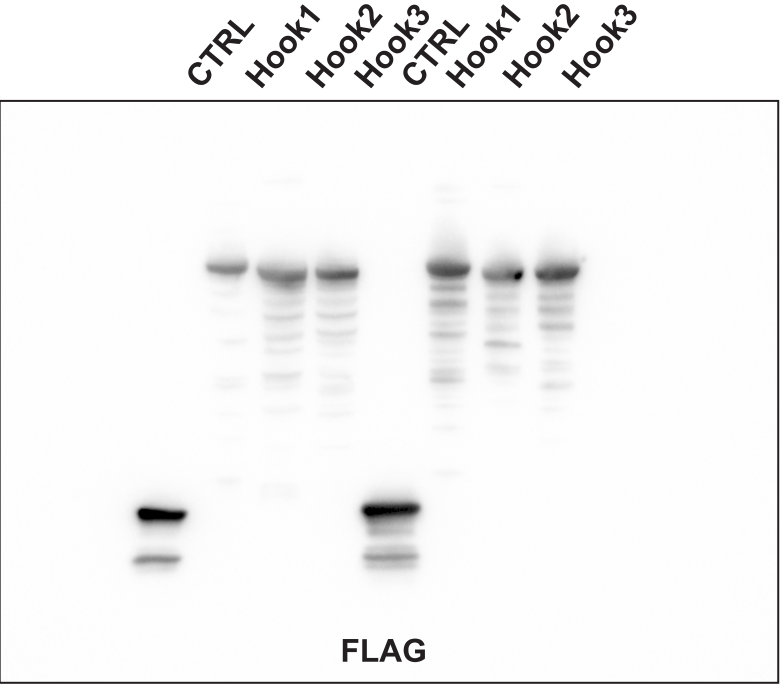

Raw image: Fig2supp1_FLAG.scn

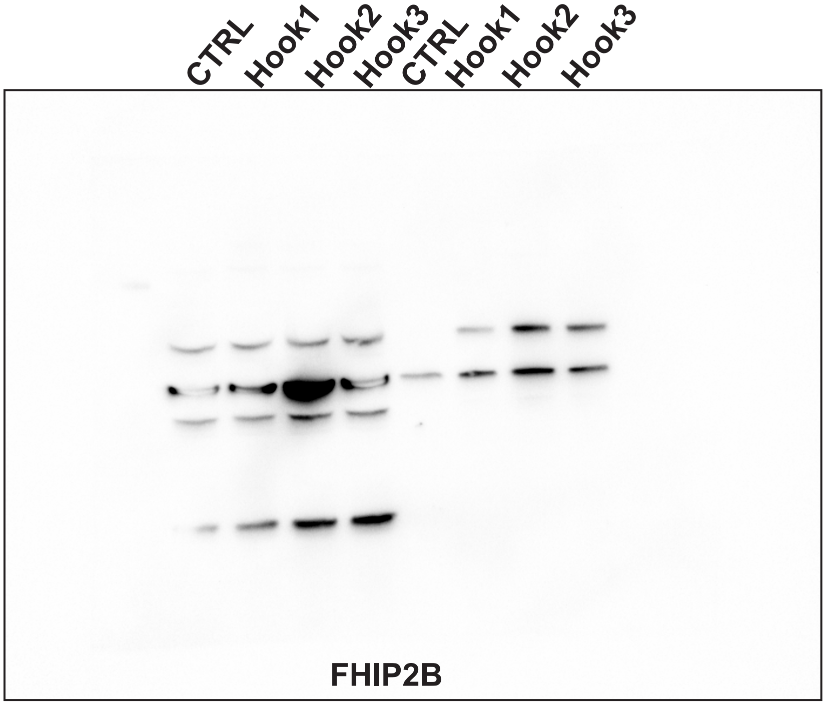

Raw image: Fig2supp1_FHIP2B.scn
