# Supplementary material for: Cytoplasmic dynein-1 cargo diversity is mediated by the combinatorial assembly of FTS–Hook–FHIP complexes
Source: eLife. 2021 Dec 9;10:e74538. doi: 10.7554/eLife.74538 (PMC8730729; doi:10.7554/eLife.74538)
Supplement: Figure 4—figure supplement 1—source data 1. — (A) Raw uncropped immunoblot images from Figure 4—figure supplement 1A (Fig4supp1A_Hook1.scn – anti-Hook1; Fig4supp1A_Hook3.scn – anti-Hook3; Fig4supp1A_Hook2.scn – anti-Hook2; Fig4supp1A_Actin.scn – anti-β-actin) probed with the indicated antibodies. Relevant lanes are marked on the images. (B) Raw uncropped immunoblot images from Figure 4—figure supplement 1B (Fig4supp1B_V5_high.scn – anti-V5; Fig4supp1B_FHIP1B_FHIP2A_high.scn – anti-FHIP1B, left side of the image and anti-FHIP2A, right side of the image; Fig4supp1B_Actin_V5_low.scn – anti-V5, left side of the image and anti-β-actin, right side of the image; Fig4supp1B_FHIP2A_low.scn – anti-FHIP2A; Fig4supp1B_FHIP1B_low.scn – anti-FHIP1B) probed with the indicated antibodies. Relevant lanes are marked on the images with numbers 1–5 as outlined in the schematic. [file elife-74538-fig4-figsupp1-data1.pdf]

A

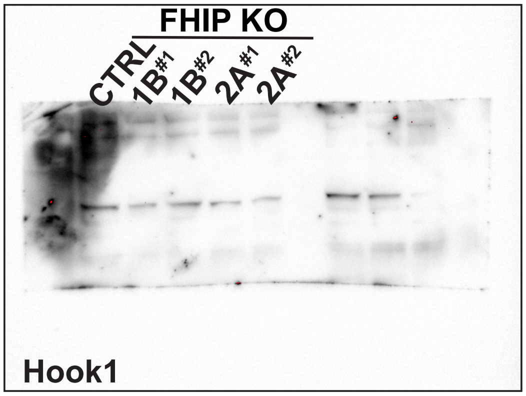

Raw image: Fig4supp1A_Hook1.scn

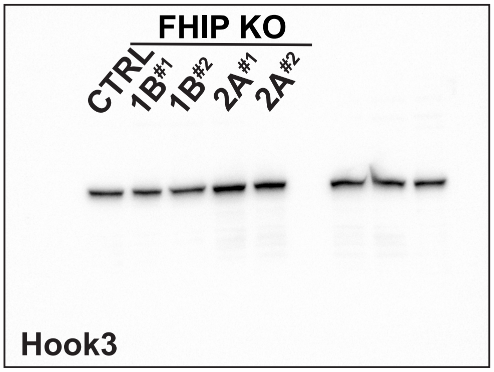

Raw image: Fig4supp1A_Hook3.scn

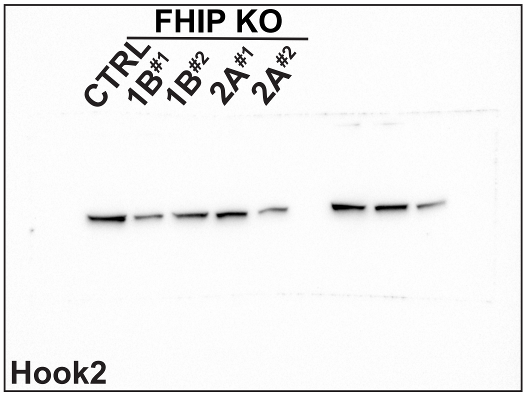

Raw image: Fig4supp1A_Hook2.scn

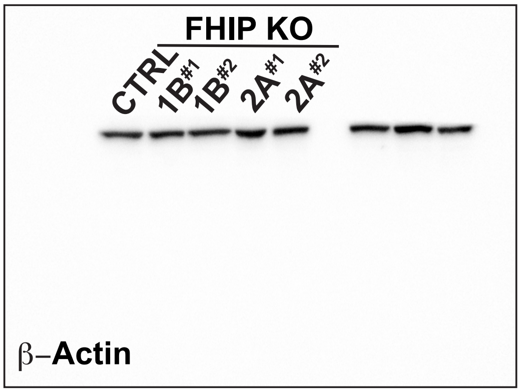

Raw image: Fig4supp1A_Actin.scn

B

CTRL	FHIP KO 1B	FHIP KO 1B	FHIP KO 2A	FHIP KO 2A	
+	+	–	+	–	tagRFP-V5
–	–	+	–	–	FHIP1B-tagRFP-V5
–	–	–	–	+	FHIP2A-tagRFP-V5
1	2	3	4	5	

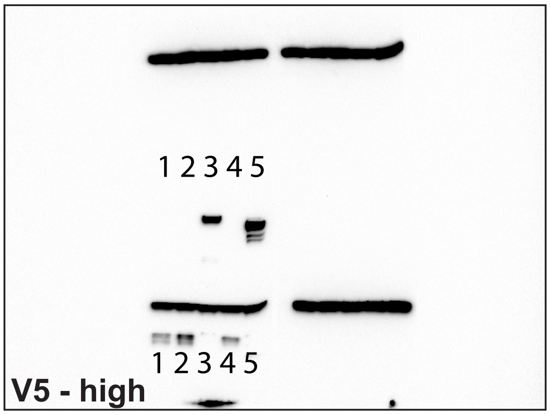

Raw image: Fig4supp1B_V5_high.scn

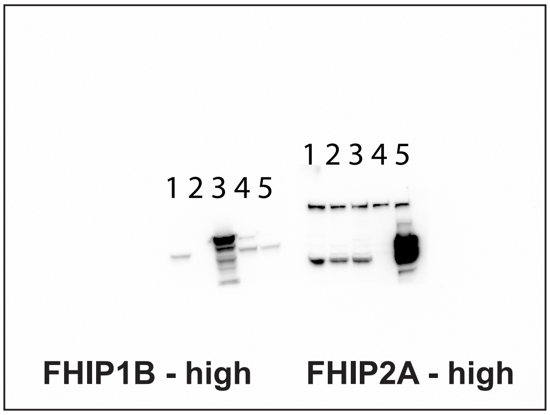

Raw image: Fig4supp1B_FHIP1B_FHIP2A_high.scn

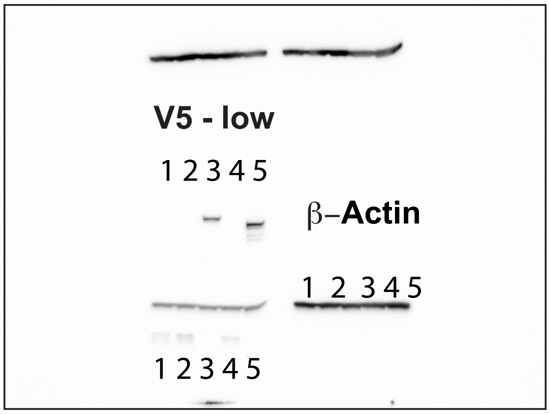

Raw image: Fig4supp1B_Actin_V5_low.scn

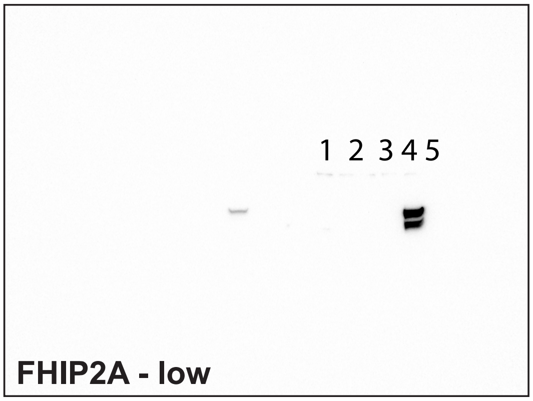

Raw image: Fig4supp1B_FHIP2A_low.scn

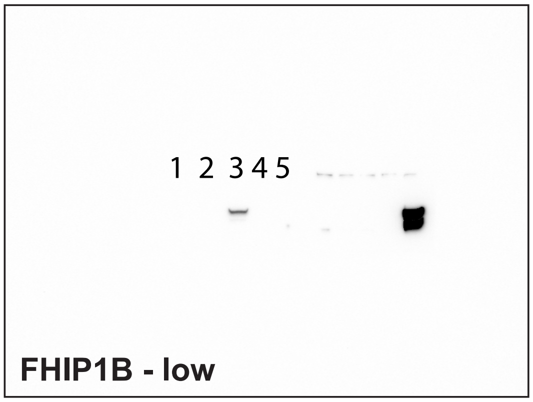

Raw image: Fig4supp1B_FHIP1B_low.scn
