# Supplementary material for: Cytoplasmic dynein-1 cargo diversity is mediated by the combinatorial assembly of FTS–Hook–FHIP complexes
Source: eLife. 2021 Dec 9;10:e74538. doi: 10.7554/eLife.74538 (PMC8730729; doi:10.7554/eLife.74538)
Supplement: Figure 5—source data 1. — (G) Raw uncropped immunoblot images from Figure 5G (Fig5G_FHIP1Binput.scn – anti-FHIP1B; Fig5G_FHIP1BIP.scn – anti-FHIP1B; Fig5G_GFP.scn – anti-GFP) probed with the indicated antibodies. Relevant lanes are marked on the images. Red lines outline the parts of the image used for figures. [file elife-74538-fig5-data1.pdf]

F

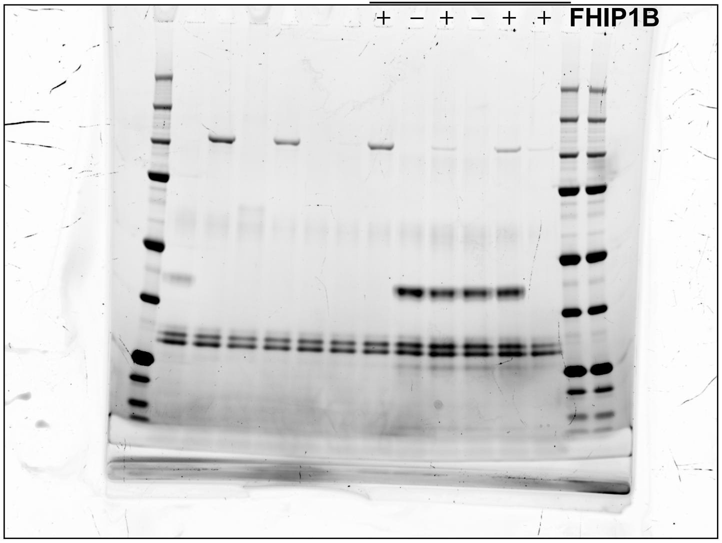

Raw image: Fig5F.scn

G

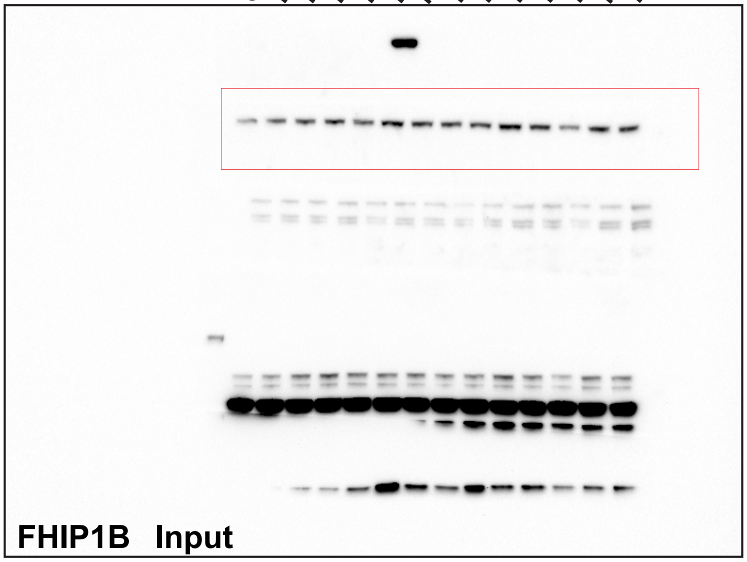

Raw image: Fig5G_FHIP1Binput.scn

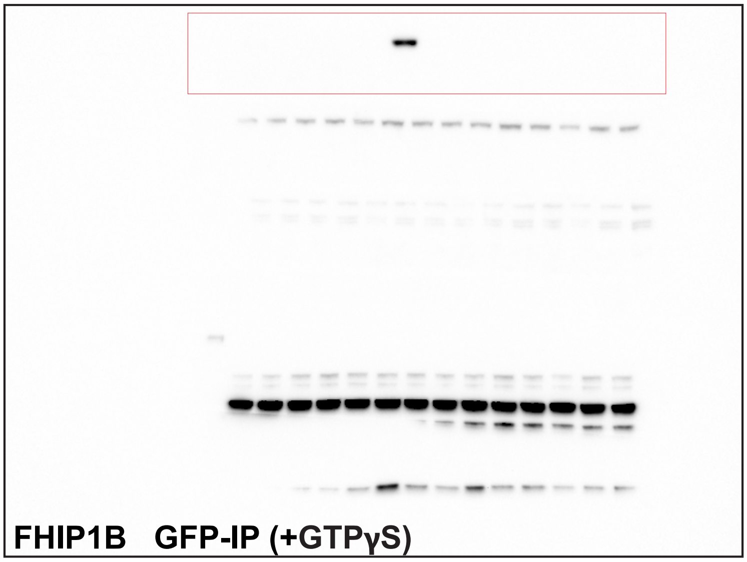

Raw image: Fig5G_FHIP1BIP.scn

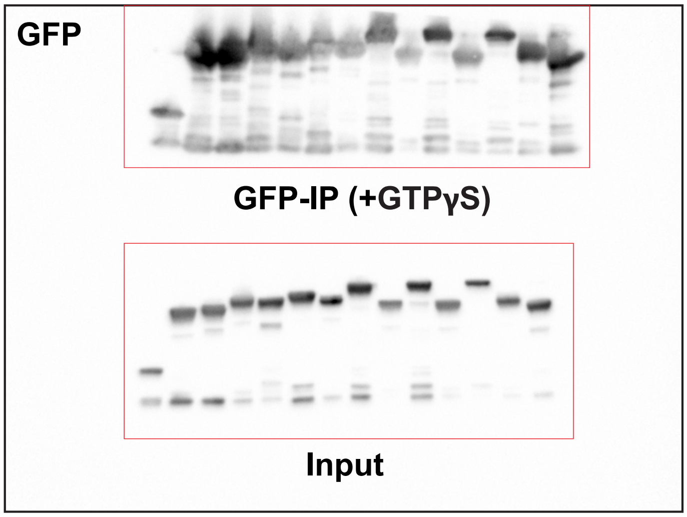

Raw image: Fig5G_GFP.scn
