# Supplementary material for: Cytoplasmic dynein-1 cargo diversity is mediated by the combinatorial assembly of FTS–Hook–FHIP complexes
Source: eLife. 2021 Dec 9;10:e74538. doi: 10.7554/eLife.74538 (PMC8730729; doi:10.7554/eLife.74538)
Supplement: Figure 6—figure supplement 3—source data 1. — Relevant lanes are marked on the images. (B) Raw uncropped immunoblot images from Figure 6—figure supplement 3B (Fig5G_FHIP1BIP.scn – anti-FHIP2A; Fig5G_GFP.scn – anti-GFP) probed with the indicated antibodies. Relevant lanes are marked on the images. Red lines outline the parts of the image used for figures. [file elife-74538-fig6-figsupp3-data1.pdf]

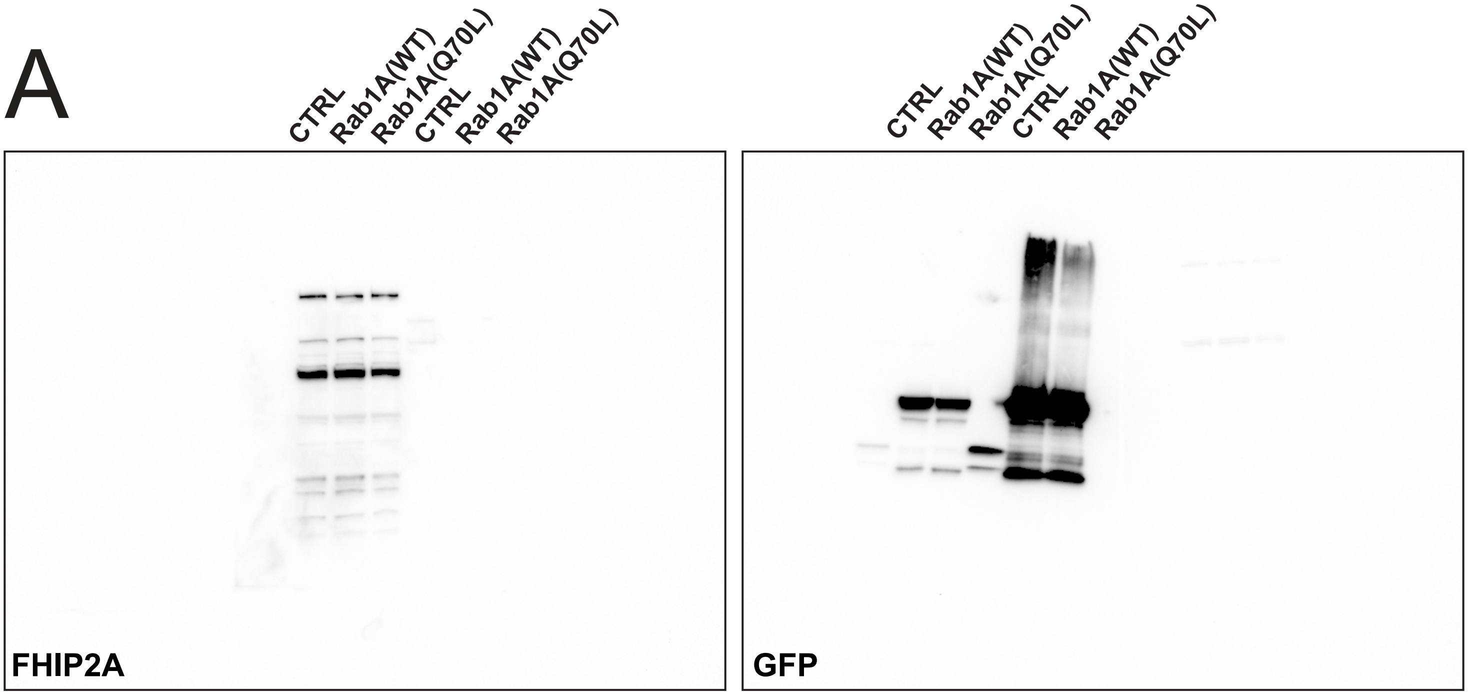

Raw image: Fig6supp3A_FHIP1B.scn

Raw image: Fig6supp3A_GFP.scn

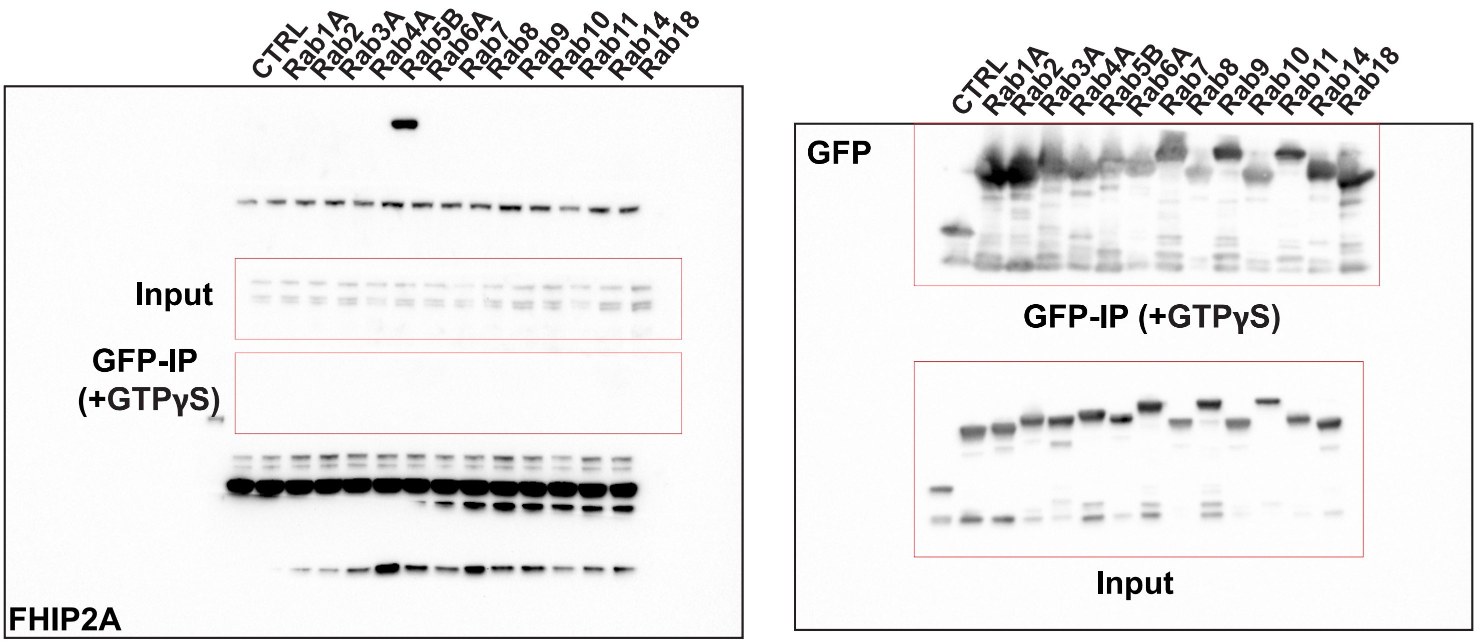

Raw image: Fig5G_FHIP1BIP.scn

Raw image: Fig5G_GFP.scn
